# LC-MS/HRMS Analysis, Anti-Cancer, Anti-Enzymatic and Anti-Oxidant Effects of *Boerhavia diffusa* Extracts: A Potential Raw Material for Functional Applications

**DOI:** 10.3390/antiox10122003

**Published:** 2021-12-16

**Authors:** Kouadio Ibrahime Sinan, Uğur Akpulat, Afaf A. Aldahish, Yasemin Celik Altunoglu, Mehmet Cengiz Baloğlu, Dimitrina Zheleva-Dimitrova, Reneta Gevrenova, Devina Lobine, Mohamad Fawzi Mahomoodally, Ouattara Katinan Etienne, Gokhan Zengin, Shafi Mahmud, Raffaele Capasso

**Affiliations:** 1 Physiology and Biochemistry Research Laboratory, Department of Biology, Science Faculty, Selcuk University, Konya 42130, Turkey; sinankouadio@gmail.com; 2 Department of Medical Biology, Faculty of Medicine, Kastamonu University, Kastamonu 37150, Turkey; uakpulat@kastamonu.edu.tr; 3 Department of Pharmacology and Toxicology, College of Pharmacy, King Khalid University, Abha 62529, Asir, Saudi Arabia; adahesh@kku.edu.sa; 4 Department of Genetics and Bioengineering, Faculty of Engineering and Architecture, Kastamonu University, Kastamonu 37150, Turkey; ycaltunoglu@kastamonu.edu.tr (Y.C.A.); mcbaloglu@kastamonu.edu.tr (M.C.B.); 5 Department of Pharmacognosy, Faculty of Pharmacy, Medical University-Sofia, 1431 Soifa, Bulgaria; dzheleva@pharmfac.mu-sofia.bg (D.Z.-D.); rgevrenova@pharmfac.mu-sofia.bg (R.G.); 6 Department of Health Sciences, Faculty of Medicine and Health Sciences, University of Mauritius, Réduit 80837, Mauritius; devinalobine@gmail.com (D.L.); f.mahomoodally@uom.ac.mu (M.F.M.); 7 Laboratoire de Botanique, UFR Biosciences, Université Félix Houphouët-Boigny, Abidjan 00225, Côte d’Ivoire; katinan.etienne@gmail.com; 8 Genetic Engineering and Biotechnology, University of Rajshahi, Rajshahi 6205, Bangladesh; s1511161112@ru.ac.bd; 9 Department of Agricultural Sciences, University of Naples Federico II, 80055 Portici, Italy

**Keywords:** *Boerhavia diffusa*, antioxidant, enzyme inhibition, anti-cancer, flavonoids, functional applications

## Abstract

*Boerhavia diffusa* is a great tropical plant and is widely used for various traditional purposes. In the present study, we examined the influence of solvents (dichloromethane, ethyl acetate, methanol and infusion (water)) on chemical composition and biological capabilities of *B. diffusa*. An UHPLC-HRMS method was used to determine the chemical characterization. The biological ability was examined for antioxidant, enzyme inhibitory and anti-cancer effects. To evaluate antioxidant effects, different chemical methods (ABTS, DPPH, CUPRAC, FRAP, metal chelating and phosphomolybdenum) were applied. With regard to enzyme inhibitory properties, cholinesterases, amylase, glucosidase and tyrosinase were used. The MDA-MB-231 breast cancer cell line was chosen to determine anticancer activity. Based on the UHPLC-HRMS analysis, 37 specialized metabolites were dereplicated and identified in the studied extracts. Results revealed the presence of 15 hydroxybenzoic, hydroxycinnamic, acylquinic acids, and their glycosides, one rotenoid, seven flavonoids, 12 fatty acids and two other glycosides. Among the tested extracts, the methanol extract showed a stronger antioxidant ability compared with other extracts. The methanol extract also showed the best inhibitory effects on tyrosinase and glucosidase. In the anti-cancer evaluation, the methanol extract showed stronger anticancer effects compared with water extract. In summary, our observations can contribute to the establishment of *B. diffusa* as a potential candidate for functional applications in the preparation.

## 1. Introduction

Over the past century, the pharmaceutical and food industries have focused more on replacing synthetic compounds with natural ones, and this fact is important in reducing consumer concerns [1]. In this sense, plants and plant extracts are gaining interest on pharmacy and market shelves [2,3,4]. Some recent reports have suggested that high consumption of vegetable derivatives is inversely related to the mortality and morbidity of some chronic diseases such as cardiovascular disease, diabetes mellitus and cancer [5]. In addition, the demand for new raw materials supply is increasing in these industries due to growing human population. Thus, the discovery of new, effective, and safe raw material is increasing day by day [6]. At this point, unexplored wild plants are considered as a promising treasure for the new raw materials. 

Cancer is rapidly becoming the leading cause of death worldwide among noncommunicable diseases. It has been diagnosed with 18.1 million new cancer cases in 2018 according to GLOBOCAN estimates, ranking breast cancer as the second most commonly diagnosed cancer [7]. Breast cancer is only curable in 70–80% of patients with early-stage and considered incurable in advanced patients with currently available therapies [8]. Excessive toxicity of conventional chemotherapies and unmet medical needs to control breast cancer demand alternative approaches. Phytochemicals, the components of the plant’s chemical core, have gained considerable interest due to their broad safety profile and potency to target multiple stages in cancer progression. There have been many reported phytochemicals and plant extracts in the literature that have preventive or treatment ability for breast cancer [9,10,11]. 

*Boerhavia diffusa* is an herbaceous and perennial medicinal plant in the Nyctaginaceae family. Various in vivo and in vitro studies confirm its leaf and root extracts have the presence of immunomodulatory, hypoglycemic, antifibrinolytic, anti-inflammatory, diuretic, hepatoprotective, antimicrobial, antioxidant, spasmolytic, and anticancer activities. This wide variety of activities have been attributed to its rich phytochemical content including flavonoid glycosides, rotenoids, steroids, alkaloids, phenolic glycosides, and lignan glycosides [12]. Its roots and leaves represent substantial differences in respect to chemical constituents and it was also reported that the same structural parts of *B. diffusa*, leaves or roots, collected from different geographical origins have different metabolite profiles [13,14]. The plant is documented to possess antioxidant [15], antimicrobial [16,17], anti-diabetic [18], immunosuppressive [19], cardioprotective [20], hepaprotective [19] and neuroprotective [21] attributes. Various categories of secondary metabolites such as alkaloids (punarnavine), rotenoids (boeravinones A–F), flavonoids, phenolics, steroids and lignan glycosides have been characterized from *B. diffusa* [13,22,23]. Anticancer activity of *B. diffusa* extracts has been shown on several in vitro and in vivo experimental designs. In the cervical cancer model, 300 µg/mL ethanolic crude root extract of *B. diffusa* has been shown to cause 30% cell death in the HeLa cell line [24]. The ethanolic root extract of *B. diffusa* has also exhibited anti-lymphoproliferative activity in the mitogen-activated human peripheral blood mononuclear cells in a dose-dependent manner with the highest inhibition rate (95.5%) at 500 µg/mL [25]. In an experimental benign prostatic hyperplasia rat model, the hydroalcoholic extract of roots of *B. diffusa* (100 mg/kg) has significantly inhibited prostate growth [26]. Prophylactic administration of the aqueous methanol extract of *B. diffusa* whole plant (0.5 mg/dose) has inhibited metastasis formation by B16F10 melanoma cells about 95% in C57BL/6 mice. In an in vitro hormone-dependent human breast cancer model, the methanolic extract of the whole plant of *B. diffusa* has shown a 46.8% reduction in cell viability in MCF-7 cells in 48 h at 320 µg/mL [27]. 

Based on afore-mentioned properties of *B. diffusa*, we aimed to examine chemical constituent profiles of several solvent extracts (dichloromethane, ethyl acetate, methanol and water (infusion)) of *B. diffusa* and analyze whether these extracts affect proliferation of MDA-MB-231 breast cancer cell line, an in vitro model of hormone-independent breast cancer, as well as antioxidant and enzyme inhibitory properties. 

## 2. Materials and Methods

### 2.1. Plant Material and Preparation of Extracts

The aerial part of *Boerhavia diffusa* L. was collected in the village of N’gbessou (district of Yamoussoukro-Côte d’Ivoire) in January 2019 and it was authenticated by the botanist Ouattara Katinan Etienne (Université Félix Houphouet Boigny, Abidjan, Côte d’Ivoire). The aerial parts were dried at room temperature for 10 days and then they were grounded by using a laboratory mill. The powdered plant materials were stored in dark condition at 4 °C. 

In the extraction stage, four solvents (dichloromethane, ethyl acetate, methanol and water) were used. To obtain organic extracts, maceration technique was used. An amount of 5 g plant materials were mixed with the solvents (100 mL) for 24 h at room temperature. Then, the mixture was filtered and then the solvents were removed by using a rotary-evaporator. Water extract was prepared as infusion technique, namely the plant material (5 g) was kept with 100 mL of boiled water for 15 min and then it was filtered and lyophilized. All extracts were stored at 4 °C until analysis. 

### 2.2. Chromatographic Separation and High-Resolution Mass Spectrometry (HRMS)

Dionex Ultimate 3000RSLC (ThermoFisher Scientific, Inc., Vantaa, Finland) with reversed phase column Kromasil EternityXT C18 (1.8 µm, 2.1 × 100 mm) column was used to separate chemical components of the tested extracts. Q Exactive Plus mass spectrometer (ThermoFisher Scientific, Inc. Vantaa, Finland) equipped with a heated electrospray ionization (HESI-II) probe (ThermoScientific, Vantaa, Finland) was used for mass analysis. All separation and mass analysis details are given in the Supplemental Materials. 

### 2.3. Assays for Total Phenolic, Flavonoid, Antioxidant and Enzyme Inhibitory Effects

TPC and TFC were determined according to previously described methods [28,29] and expressed as mg GAE/g (TPC) and mg RE/g (TFC). DPPH, ABTS, CUPRAC and FRAP were performed as in [28,29], with the results presented as mg TE/g. MCA and PBD were carried out as mentioned in [28,29], with the data provided as mg EDTAE/g (MCA) and mmol TE/g (PBD). AChE, BChE, tyrosinase, amylase and glucosidase inhibition methods were detailed in [28,29]. The anti-enzymatic activities were expressed as mg GALAE/g in AChE and BChE assays, mg KAE/g d.w. in tyrosinase assay and mmol ACAE/g d.w. in amylase and glucosidase assays. 

### 2.4. Cell Culture

#### 2.4.1. Cell Culture Reagents 

DMEM cell culture media, penicillin/streptomycin, fetal bovine serum (FBS), human insulin, non-essential amino acid solution (NEAA), 0.25% trypsin-EDTA solution and thiazolyl blue tetrazolium bromide powder (MTT) were purchased from Sigma-Aldrich (Sigma-Aldrich, Saint Louis, MO, USA). 

#### 2.4.2. Preparation of *B. diffusa* Extracts for Cell Culture

Water and methanol extracts of *B. diffusa* were dissolved in 1× PBS at the concentration of 10 mg/mL as the stock solution and filtrated with 0.22 μm filter membrane. Stock solutions were stored at a −20 °C freezer until further use. For all experiments, working dilutions of the extracts were prepared by diluting the stock solutions with the complete culture medium.

#### 2.4.3. Cell Culture Maintenance

Triple-negative MDA-MB-231 human breast adenocarcinoma cells (obtained from Bogazici University, Department of Molecular Biology and Genetics) were maintained in Dulbecco’s Modified Eagle’s Medium (DMEM) supplemented with 10% FBS, 0.01 mg/mL human insulin, 1% NEAA solution, and 0.1% penicillin/streptomycin at 37 °C in a 5% CO_2_ humidified incubator. To avoid over 80% confluency, cells were routinely subcultured by washing 1× PBS and then trypsinizing with 0.25% trypsin-EDTA solution. 

#### 2.4.4. In Vitro Cytotoxicity Assay

In vitro cell survival analyzes were performed by MTT assay as described before with slight modifications [30]. Cells at the exponential growth phase were cultured on 96-well plates at the seeding density of 1.0 × 10^4^ cells per well. After 24 h, the cells were incubated with various doses of water and methanol extracts of *B. diffuse* at the concentrations of 100, 200, 400, 600, and 800 µg/mL in the final volume of 100 µl of complete growth medium for 24 and 48 h. After each incubation period, the growth medium was aspirated and replaced with MTT medium supplemented with 5mg/mL MTT reagent and 0.5% FBS in DMEM and incubated for 4 h at 37 °C. The medium was removed, and formazan crystals formed were dissolved in DMSO. The plates were read in a microplate reader (Multiskan Go, Thermo Scientific, USA) operating at 570 nm. The GraphPad Prism 7 software (San Diego, CA, USA) was used to calculate the half-maximal inhibitory concentration (IC_50_) value of each sample using “log (inhibitor) vs. normalized response-variable slope analysis function”. MTT assays were performed on both biological and experimental replicates by handling different passages of the cells and triplicate wells for each concentration respectively to obtain statistically significant results.

### 2.5. Data Analysis

Means of triplicate analysis were computed and data was given as mean ± SD. ANOVA statistical analysis was performed for comparison between samples. A difference was considered to be statistically significant when *p* < 0.05 and Tukey’s post hoc test was conducted. The variation between the solvents was assessed through Principal component (PCA) analysis and heatmap, in consideration of all bioactivities. The statistical analysis was performed using R software v. 3.6.2.

## 3. Results and Discussion

### 3.1. Phytochemical Composition

**Figure 1:**
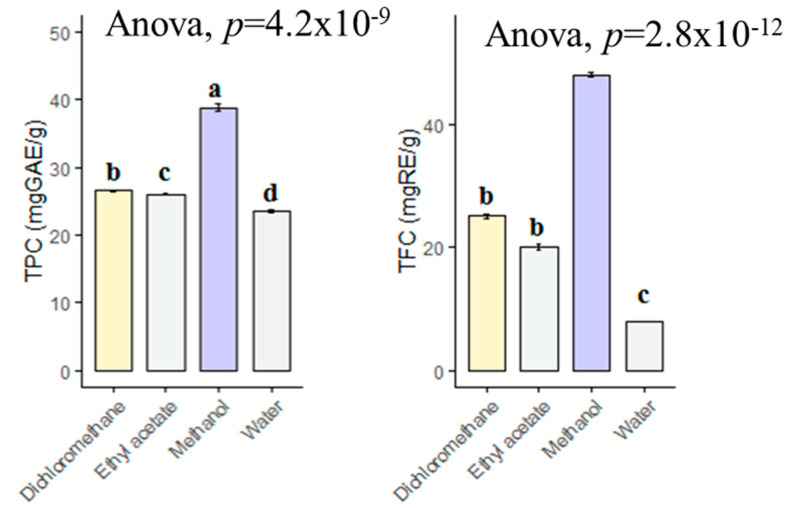
Total phenolic and flavonoids contents of tested extracts. GAE: Gallic acid equivalent; RE: Rutin equivalent. a–d bar wise values with same superscripts of this type indicate no significant difference among extracts (*p* > 0.05).

The choice of solvent is one of the most important steps in the preparation of plant extracts. In this context, phytochemists have used various solvents to determine which solvent is the best and to determine the type of phytochemicals in the plants. In the present study, the methanol extract contained the highest total phenolic content with 38.85 mg GAE/g, followed by dichloromethane (26.61 mg GAE/g), ethyl acetate (26.06 mg GAE/g) and infusion (23.55 mg GAE/g) (Figure 1). However, the levels in dichloromethane and ethyl acetate extracts were statistically similar (*p* > 0.05). With regard to total flavonoid content, the extracts were in same order (methanol > dichloromethane > ethyl acetate > infusion). Taken together, we have suggested that methanol is a good solvent for other applications with *B. diffusa*. In the literature, we observed different values for total phenolic and flavonoid contents of *B. diffusa* extracts. For example, Gophane and Khobragade [31] were investigated different extracts of *B. diffusa* and the values ranged from 85 mg GAE/g (water extract) to 155.35 mg GAE/g (ethanol). Additionally, the highest flavonoid level was provided by ethanol extract (75.19 mg RE/g) in their study. In another study conducted by Irshad et al. [32], the total phenolic and flavonoid contents in the methanol extract of *B. diffusa* were reported as 92.78 mg GAE/g and 34.38 mg QE (quercetin equivalent)/g, respectively. The differences might be explained by geographical factors (altitude, soil, etc.), pedoclimatic conditions (rainfall, etc.) and extraction methods (maceration, soxhlet, ultrasonication, etc.). In the latter scenario, spectrophotometric methods alone are not insufficient to determine the chemical composition of plant extracts, since the used reagents in the spectrophotometric methods cannot be reduced by just as specific phytochemical group. Thus, further chromatographic techniques such as LC-MS, LC-MS/QTOF or NMR have to characterize the components in the plant extracts. 

### 3.2. Dereplication and Annotation of Specialized Metabolites in Boerhavia diffusa Extracts

**Figure 2:**
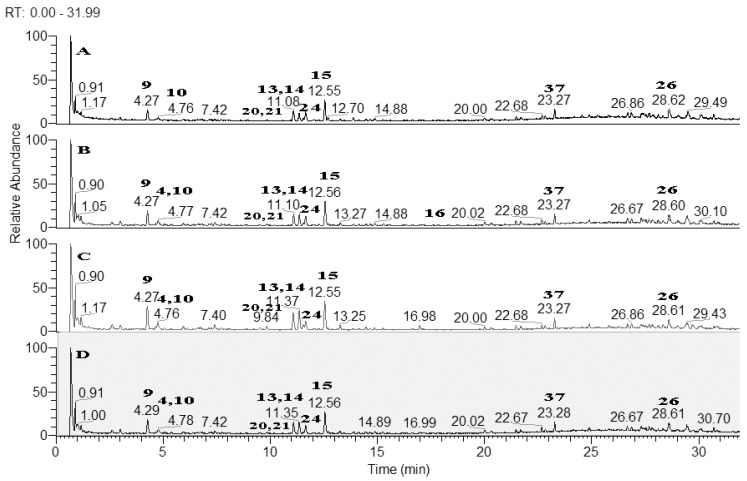
Total ion chromatograms (TIC) in negative ion mode of the studied *Boerhaavia* extracts; A-Ethyl acetate extract; B-MeOH extract; C-Dichloromethane extract; D-Infusion with water. For peaks annotation see Table 1.

**Figure 3:**
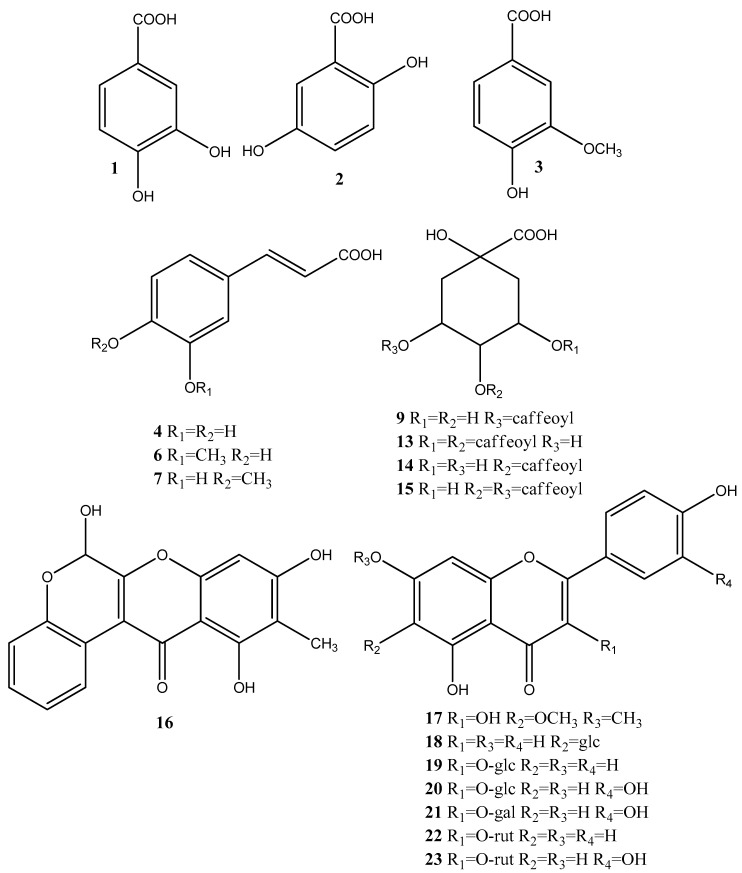
Structure of the bioactive metabolites, found in the studied *Boerhaavia* extracts; glc = glucose, gal = galactose; rut = rutinose.

Based on the accurate masses, MS/MS fragmentation patterns, relative abundance of the fragment ions, and comparison with reference standards and literature data, 37 specialized metabolites were dereplicated and identified in the assayed extracts. Results revealed the presence of 15 hydroxybenzoic, hydroxycinnamic, acylquinic acids, and their glycosides, 1 rotenoid, 7 flavonoids, 12 fatty acids and 2 other glycosides. Total ion chromatograms in negative ion mode as well as the major compounds in the studied extracts were presented in Figure 2. The structure of some metabolites, found in the studied *Boerhaavia* extracts, were presented in Figure 3.

Hydroxybenzoic (1–3), hydroxycinnamic acids (4, 6, and 7), their glycosides (8, 11 and 12) and quinic acid (5) were identified based on the comparison with reference standards and literature data [33] (Table 1). 

The acylquinic acids dereplication was based on the conformity with the structure-diagnostic hierarchical keys for chlorogenic acids identification proposed by [34] and later developed by [35], as well as literature data acquired by hybrid Q-Orbitrap mass spectrometry [36]. Thus, two mono-acylquinic (9–10), and three di-acylquinic (13–15) was identified in the studied extracts (Table 1). 

Compound 16 [M-H]^−^ at *m*/*z* 311.055 gave fragment ions at *m*/*z* 283.0589 due to loss of CO and Retro-Diels-Alder (RDA) ions at *m*/*z* 133.027 [^0.3^A]^−^ and 109.027 [^0.4^A]^−^. Thus, 16 was tentatively assigned to the rotenoid boeravinone B, previously found in *B. diffusa* [37]. Compound 17 [M-H]^−^ at 329.066 showed fragment ions at *m*/*z* 314.043 and 299.019 due to successive loss of CH_3_ followed by the transition *m*/*z* 299.018–271.0225 [M-H-2CH_3_-CO]^−^. Based on the RDA fragment ions at *m*/*z* 180.988 [^0.4^A-CH3]^−^ and 151.002 [^1.3^A]^−^, 17 could be related to eupalitin, previously identified in *B. diffusa* [37]. In (−) ESI-MS/MS 18 ([M-H]^−^ at *m*/*z* 431.098) afforded a base peak at *m*/*z* 431.098 together with the abundant fragment ion (70.53%) at *m*/*z* 311.055 [(M-H)−120]^−^ (^0.2^X^−^) and prominent ion at *m*/*z* 283.061 (^0.2^X^−^−CO), both indicating *C*-glycoside [33]. Thus, compounds 18 were ascribed as apigenin-*C*-6 glucoside (isovitexin) (Table 1).

MS/MS fragmentation pathways of the flavonol *O*-glycosides 19–23 yielded neutral mass losses of 162.053 and 308.112 Da consistent with hexose and rutinose, supported by the RDA cleavages of the flavonoid skeleton [36]. Compounds 20, 21 and 23 showed a fragment ion at *m*/*z* 301.035, together with RDA ions at *m*/*z* 151.002 [^1.3^A]^−^, 121.027 [^1,2^B]^−^ and 107.012 [^0.4^A]^−^, suggesting the presence of the quercetin core well [33]. Compounds 19 and 22 showed a indicative ion at *m*/*z* 285.038, and could be attributed to kaempferol glycosides. Isovitexin (18), isoquercitrin (20), hyperoside (21), kaempferol 3-*O*-rutinoside (22) and ruin (23) were identified by comparison with the retention times in UHPLC-HRMS and MS/MS fragmentation fingerprints of reference standards (Table 1).

In (−) ESI-MS/MS of 24 [M-H]^−^ at *m*/*z* 187.096, the concomitant losses of H_2_O (−18) and CO_2_ (−44), yielded a base peak at *m*/*z* 125.095 ([M-H-CO_2_-H_2_O]^−^ and characteristic ion at *m*/*z* 97.064 [M-H-2CO_2_]^–^. Based on comparison with the literature data, 24 could be related to the saturated dicarboxylic acid-nonanedioic acid (azelaic acid) (Table 1) [38]. In the (−) ESI-MS/MS spectrum of 25 (C_12_H_20_O_4_), a base peak at *m*/*z* 183.138 [M-H-CO_2_]^−^ and a fragment ion at *m*/*z* 165.127 (C_11_H_17_O) indicated the presence of double bond at C-2. Dereplication of 25 as dodec-2-endioic acid (traumatic acid) was based on the literature data [39]. In the same manner, based on accurate masses, fragmentation patterns, and comparison to literature data, two monounsaturated (26 and 31) and six polyunsaturated (26, 28–30, and 32–33) free fatty acids were tentatively dereplicated in *B. diffusa* extracts (Table 1) [40,41].

Compound 36 [M-H]^−^ at *m*/*z* 409.208 gave fragment ions at *m*/*z* 277.166 and 161.043 corresponding to the subsequent loss of pentose (−132.05 Da) and heptanol (−116.12 Da), respectively. In addition, fragment ions at 71.012, 89.0229, and 101.022 could be attributed to cross-ring cleavages of deprotonated hexose [42]. Thus, 36 could be ascribed to heptanol pentosyl-hexoside, previously found in *Cosmos caudatus* extracts (Table 1) [43]. MS/MS fragmentation pathway of 37 revealed consecutive loss of hexose at *m*/*z* 631.385 and hexuronic acid at *m*/*z* 455.358 and corresponded to ursolic acid hexuronyl-hexoside (Table 1).

### 3.3. Antioxidant Property

**Figure 4:**
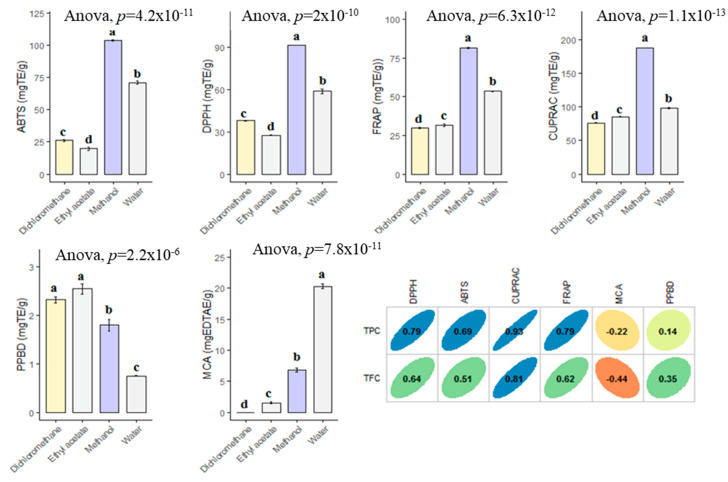
Antioxidant properties of the tested extracts and their correlation with TPC and TFC. TE: Trolox equivalent; EDTAE: EDTA equivalent. a–d bar wise values with same superscripts of this type indicate no significant difference among extracts (*p* > 0.05).

**Figure 5:**
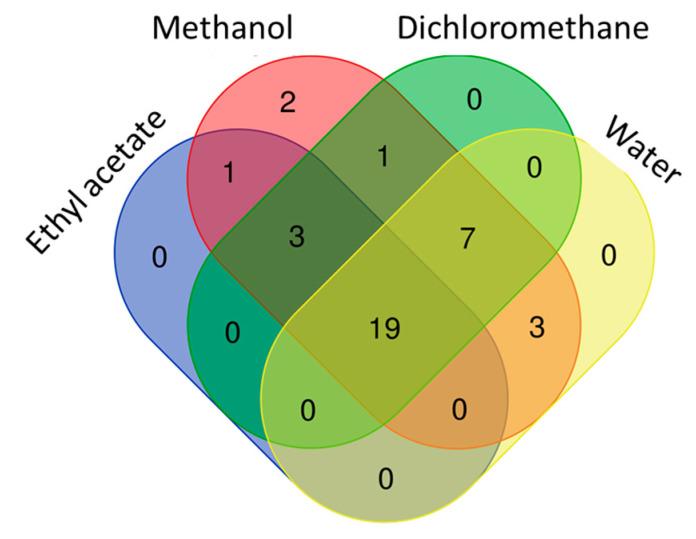
Venn diagram showing number of common compounds in the tested extracts.

For past decade, the terms of “antioxidant” has been one of the most popular topics in scientific research. Many researchers are looking for new and safe sources of antioxidants. Antioxidants are considered to be a strong protective shield against oxidative stress, which is responsible for the progression of chronic and degenerative diseases. Plant or plant products are the main sources of natural antioxidant in this regard. In the current work, the antioxidant properties of *B. diffusa* extracts were demonstrated by using different chemical assays including radical quenching, reducing power and metal chelating (Figure 4). In free radical scavenging assays (ABTS and DPPH), the methanol extract showed the strongest ability (DPPH: 91.62 mg TE/g and ABTS: 103.56 mg TE/g), followed by infusion (DPPH: 58.89 mg TE/g and ABTS: 71.04 mg TE/g), dichloromethane (DPPH: 37.99 mg TE/g and ABTS: 26.09 mg TE/g) and ethyl acetate (DPPH: 27.61 mg TE/g and ABTS: 19.84 mg TE/g). Apparently, all extracts had different radical scavenging properties. In accordance with radical scavenging assays, the best reducing abilities were found in the methanol and infusion extracts. The observations could clearly be explained with the higher concentration of the level of total phenolic compounds in the extracts. In this regard, Pearson’s correlation analysis also showed a strong relationship between total phenolics and the radical scavenging and reducing power assays. In addition, the methanol extract contained more compounds when compared with other extracts and Figure 5 summarized the numbers of identified compounds in the tested extracts. Transition metals play an important role in Fenton and Haber–Weiss reactions that generate hydroxyl radicals. At this point, metal ion chelation is considered to be another mechanism in the antioxidant mechanisms. In metal chelating assay, the infusion showed the best ability with 20.32 mg EDTAE/g, followed by methanol (6.80 mg EDTAE/g) and ethyl acetate (1.55 mg EDTAE/g). However, dichloromethane was not active in the assay. The phosphomolybenum assay is one of the most popular antioxidant assays in recent times because it is simple, inexpensive and does not require special equipment. In the assay, Mo (VI) is reduced by antioxidant compounds to Mo (V) and this conversion could be provided by both phenolic and non-phenolic antioxidants. In contrast to other antioxidant assays, ethyl acetate (2.55 mmol TE/g) was the best, followed by dichloromethane (2.32 mmol TE/g), methanol (1.80 mmol TE/g) and infusion (0.74 mmol TE/g). As can be seen in Figure 4, we observed weak correlation values between total phenolic content and phosphomolybenum, as well as metal chelating. This fact could be explained by the presence of non-phenolic antioxidants such as tocopherols, carotenoids or vitamin C. In addition to this approach, the conflicting results between phosphomolybdenum and other antioxidant assays (especially free radical scavenging assays) could be related to the presence of other reducing agents including peptides or sugars. In previous studies, in agreement with our approaches, some peptides showed a reducing potential in the phosphomolybdenum assay [44,45]. 

In the literature, several authors have reported antioxidant properties of *B. diffusa* extracts and they have found that the antioxidant properties are depended on the extraction solvents used. Gophane and Khobragade [31] reported that the DPPH radical scavenging abilities of the tested extracts were in the following order: ethanol > acetone > ethyl acetate > aqueous. This order was also reported as butanol > ethanol > ethyl acetate > chloroform in another study conducted by Khalid et al. [46]. A previous study [47] found that methanol extract was the most active against DPPH radicals when compared to ethanol and water extracts. As a further finding, the methanol extract contained more compounds compared to other extracts and thus the observed antioxidant abilities for methanol could be explained with the presence of these compounds. For example, boeravinone B was only detected in the methanol extract and boeravinone derivatives were reported as an antioxidant compounds in previous studies [48,49,50]. In addition to this compound, other compounds such as caffeic acid, kaempferol, or rutin could contribute the reported antioxidant properties for the methanol extract [51,52,53]. 

### 3.4. Enzyme Inhibitory Property

In the last century, the prevalence of some diseases (Alzheimer’s disease, diabetes mellitus, obesity, etc.) is increasing day by day and therefore, we need effective treatment strategies. In this sense, enzymes are considered to be effective modulators and the inhibition of some enzymes is closely related to the regulation of pathological events in the diseases mentioned. At this point, some enzymes are pharmaceutical targets to alleviate the symptoms observed in the diseases. For example, acetylcholinesterase is a pharmaceutical target in the treatment of Alzheimer’s disease and its inhibition could help to increase cognitive functions in Alzheimer’s patients [54]. As another example, amylase and glucosidase are important players in diabetes mellitus and their inhibition is closely related to the control of blood glucose level [55]. Overall, enzyme inhibitory agents are gaining in importance in the medicinal and pharmaceutical fields. Although some enzyme inhibitors are made chemically, most have unpleasant side effects [56,57]. We therefore need novel and effective inhibitors from natural sources, especially from plants. 

**Figure 6:**
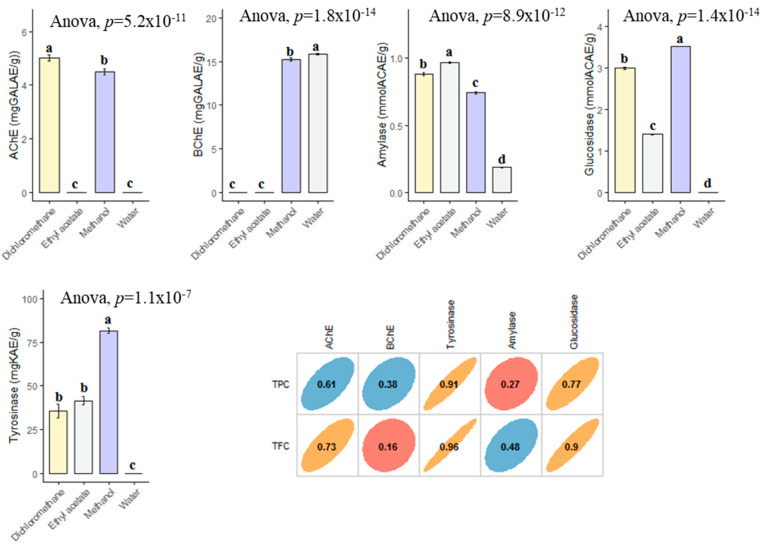
Enzyme inhibitory properties of the tested extracts and their correlation with TPC and TFC. GALAE: Galatamine equivalent; KAE: Kojic acid equivalent; ACAE: Acarbose equivalent. a–d bar wise values with same superscripts of this type indicate no significant difference among extracts (*p* > 0.05).

In the current study, the enzyme inhibitory effects of *B. diffusa* extracts were tested against cholinesterases (AChE and BChE), tyrosinase, amylase and glucosidase. In AChE inhibitory assay, two extracts (dichloromethane and methanol) were active on the enzyme and the best action was determined in dichloromethane (5.01 mg GALAE). With regard of BChE, infusion and methanol extracts were active, and the best action was recorded in infusion. The highest tyrosinase inhibitory effect was provided by the methanol extract with 81.57 mg KAE/g, followed by ethyl acetate (41.55 mg KAE) and dichloromethane (35.62 mg KAE/g) (Figure 6). However, the water extract was not active on tyrosinase. Similar to tyrosinase, the strongest glucosidase inhibitory effect was determined in the methanol extract. Lastly, the amylase inhibitory effect can be ranked as ethyl acetate > dichloromethane > methanol > infusion. When all results were evaluated with together, the obtained enzyme inhibitory results may be linked to the chemical composition of the tested extracts. Although AChE, tyrosinase and glucosidase inhibitory effects of the extracts were well correlated to their total phenolic level, BChE and amylase were not directly linked to total phenolic level. The contradictory results were also observed in the literature and this phenomena could be explained by the complex nature and possible interactions of phytochemicals. The observed enzyme inhibitory abilities of methanol extract could be the presence of boeravinone B and interactions between the compounds and others (rutin, caffeic acid, kaempferol and gallic acid, etc.). In an earlier study conducted by Ademosun et al. [58], rutin exhibited remarkable inhibition abilities against cholinesterase. Similarly, caffeic acid had a good anticholinesterase potential in a previous study [59]. In addition to the cholinesterase potential, these compounds showed good antidiabetic potential with the important amylase and glucosidase inhibitory abilities [60,61,62]. To the best of our knowledge, there are very limited studies on the enzyme inhibition properties of *B. diffusa* [47,63,64,65] and so our findings could provide a powerful cornerstone on the seeking for safe enzyme inhibitor road.

### 3.5. Principal Component Analysis

**Figure 7:**
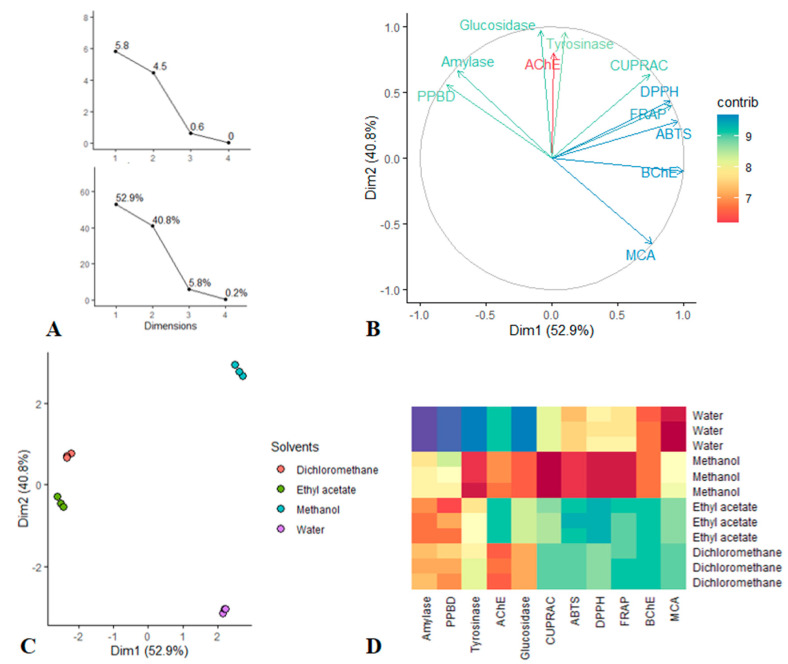
(**A**–**C**): Principal Component Analysis; (**A**) Eigenvalue and Percentage of variability explained by each dimensions. (**B**) circle of correlation showing the relation between the bioactivities and Dim 1, Dim 2. (**C**) Scatter plots displaying the distribution of solvents on the factorial plan Dim 1 vs. Dim 2. (**D**) Heatmap showing bioactivities variation between the solvents.

PCA was done to view the dissimilarity tendency between extraction solvents by taking account together the evaluated antioxidant and enzyme inhibitory activities. Figure 5 presented all of graphical results; viewing the first Figure 7A, it was found that only the first two dimension of PCA had the largest eigenvalues (larger than 1) and enclosed together more than 93% of the total variance. Thus according to Kaiser Criterion, Dim 1 and Dim 2 were retained for further investigation. The bioactivities describing the retained dimension was depicted in Figure 7B. Indeed Dim 1 which resumed 52.9% of the total variance, was significantly determined by BChE, FRAP, ABTS and DPPH while Dim 2 that synthetized 40.8% of the total variance, was predominantly determined by glucosidase, tyrosinase and AChE. Thereafter, the scatter plot was examined, as can be seen, the less polar solvents, i.e., ethyl acetate and dichloromethane closed together and they were separated from the polar solvents, i.e., methanol and water. In particular, looking at the Heatmap, ethyl acetate and dichloromethane were characterized by a highest total antioxidant and amylase inhibition capacities. Similarly, methanol exhibited strongest antioxidant properties, anti-tyrosinase and anti-glucosidase activities when compared with water which provided excellent anti-BChe and metal chelating abilities. 

In recent work, Kanagavalli et al. [66] demonstrated that the extraction solvents had a strong impact on the yields of phytochemical compounds as well as the antioxidant activity of *B. diffusa*, thus corroborating our findings. According to the authors, these variation in the yields of bioactive compounds and the antioxidant activity could be attributed not only to the difference in the chemical structure of plant phytochemical compounds but also to the nature and polarity of solvents used. In fact, the extraction of molecules from herbals could be affected by the chemical structure, the dielectric constant or organic solvents. Overall, as regards antioxidant properties methanol was found to be the best solvent, following by the water, which suggests the richness of *B. diffusa* aerial part in polar substances. Our result was in adequacy with that obtained by Bowyer et al. [67], who reported that methanol and water extracted the highest yield of phytochemicals from *Paramignya trimera* root and exhibited the strongest antioxidant activity.

### 3.6. Evaluation of the Anticancer Activity of B. diffusa Extracts

**Figure 8:**
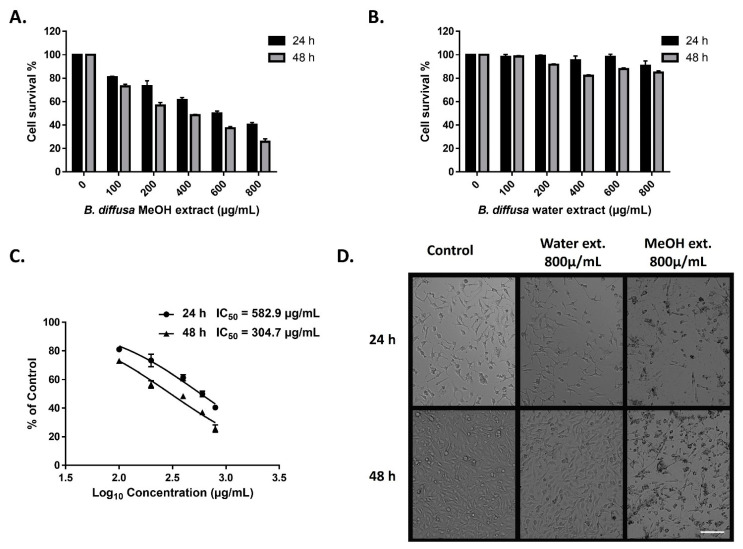
Anticancer activity of methanol and water extracts of *B. diffusa* on MDA-MB-231 breast cancer cells. Cell survival rates after 24 h and 48 h treatment by methanolic (**A**) and water (**B**) extracts. IC_50_ values of the methanolic extract after 24 h and 48 h treatments (**C**). Representative images for the morphological appearance of the treated cells (**D**). Data represent the means ± SD (*n* = 3). The scale bar is 100 µm.

The cytotoxic activity of the extracts of aerial part of *B. diffusa* on triple-negative MDA-MB-231 breast cancer cells was determined by the MTT cell viability test. The cells were treated for 24 and 48 h with variable doses of methanol and water extracts. The methanolic extract reduced viable cell numbers in all doses after both periods (Figure 8A). Only a minor reduction in cell numbers was observed in the cells treated by over 200 µg/mL water extract for 48 h (Figure 8B). Morphological observation of the cells was also supported differences between anticancer abilities of the extracts in the applied doses and time durations (Figure 8D). Treatment by the methanolic extract represented anticancer activity in a dose and time dependent manner (Figure 8A,C). While IC_50_ values were 582.9 µg/mL and 304.7 µg/mL for 24 h and 48 h treatments respectively in the methanolic extract-treated cells, the IC_50_ values of water extract could not be calculated due to cell viability was higher than 50% for both 24 h and 48 h incubation (Figure 8C). 

Based on the observation of the cytotoxic activity of the methanolic extract on hormone-independent breast cancer cells, phytochemical constituents that may be responsible for this behavior were investigated in the literature. Boeravinone B belonging to the rotenoid category is the chemical marker for *B. diffusa* [12]. It has been shown that Boeravinone B causes decreased cell survival and apoptosis in SW-620, H-29, and HTC-116 colon cancer cell lines by internalizing ErbB2 and EGFR receptors [68]. Caffeic acid is a hydroxycinnamic acid and found in fruits, coffee, and vegetables. Caffeic acid has been found to induce cell cycle arrest and apoptosis in MDA-MB-231 breast cancer cells and also reduce cell survival and activate apoptosis in MCF-7 breast cancer cells [69,70]. Ferulic acid is a widely distributed phenolic constituent found in plant cell walls. The cytotoxic activity of ferulic acid has been shown on three different breast cancer cell lines. In MDA-MB-231 cells, ferulic acid has decreased cell viability by inducing apoptosis and suppressed metastasis by reversing epithelial-mesenchymal transition [71]. In the MCF-7 cells, ferulic acid has reduced viable cell numbers and new DNA synthesis by inhibiting the EGFR receptor [72]. Other than the human breast cancer cell lines, ferulic acid has also inhibited the growth of 4T1 mouse breast cancer cells [73]. Isoquercitrin, a naturally occurring dietary flavonoid is widely found in tea, onion, and currant leaves. Isoquercitrin has been shown to induce the mitochondrial-mediated apoptosis pathway via the inhibition of lysine-specific demethylase 1 (LSD) in the MDA-MB-231 breast cancer cells, which over-express LSD, a histone-modifying enzyme [74]. Rutin is a quercetin glycoside found in a wide variety of plants, especially in invasive plant species. It has been found that rutin enhances chemosensitivity to cyclophosphamide and methotrexate in MDA-MB-231 and MCF-7 breast cancer cell lines by reversing multidrug resistance via inhibition of P-gp and BCRP pumps [75]. Chlorogenic acid is a polyphenol compound that is particularly abundant in the human diet, such as coffee and some fruits including berries, pears, and apples. Chlorogenic acid has been shown to inhibit proliferation, induce apoptosis, and suppress migration of human MDA-MB-231 and MDA-MB-453 cells, and murine 4T1 breast cancer cells by impairing the NF-κB/EMT signaling pathway [76]. In 4T1 breast cancer cells, it has been revealed that chlorogenic acid induces apoptosis via p53, Bax, Bcl-2, and caspase-3 signaling pathways [77,78]. Traumatic acid is an oxidative derivative of unsaturated fatty acids and belongs to cytokinin plant hormones. Traumatic acid has shown to decrease cell proliferation and viability and induce apoptosis by influencing lipid peroxidation in MCF-7 breast cancer cells [79]. 

As above mentioned, literature-searching revealed that methanolic extract of the aerial part of *B. diffusa* has many secondary metabolites, which decrease the number of several breast cancer cell types by influencing various biological pathways. Several or all of those metabolites likely contribute to here presented anticancer activity of the methanolic extract of *B. diffusa* synergistically. In addition, as distinct from the study that revealed the antiproliferative activity of the methanolic whole plant extract *of B. diffusa* on hormone-dependent MCF-7 breast cancer cells [27], the present study shows the cytotoxic activity of the methanolic aerial part extract of *B. diffusa* on hormone-independent MDA-MB-321 breast cancer cells.

## 4. Conclusions

The current study reports the metabolite profile of the aerial part of *B. diffusa* for the first time and introduces new phytochemicals belonging to that part of the plant to the literature. In the chemical characterization, phenolic acids and flavonoids were main constituents. Generally, the methanol extract exhibited stronger antioxidant abilities with a high level of phenolics and flavonoids compared with other tested extracts. With regard to enzyme inhibitory effects, different results were observed, but again the methanol extract was the most active on tyrosinase and glucosidase. The study also reveals the anticancer activity of methanolic extract of *B. diffusa* on hormone-independent breast cancer cells. Given these findings, *B. diffusa* could be a potential candidate for safe and effective source of bioactive components in the preparation of multi-directional applications to combat oxidative stress and cancer.

## Figures and Tables

**Table 1:** Specialized metabolites in *Boerhaavia* extracts.

No.	Identified/Tentatively Annotated Compound	Molecular Formula	Exact Mass [M-H]^−^	Fragmentation Pattern in (−) ESI-MS/MS	t_R_ (min)	Δ ppm	Distribution
Hydroxybenzoic, Hydroxycinnamic, Acylquinic Acids, and Derivatives							
1	protocatechuic acid ^a^	C_7_H_6_O_4_	153.0179	153.0179 (17.87), 109.0278 (100)	2.01	−3.554	1, 2, 3, 4
2	gentisic acid ^a^	C_7_H_6_O_4_	153.0178	153.0179 (78.01), 135.0071 (28.94), 109.0278 (100)	4.98	-3.554	1, 2, 3, 4
3	vanillic acid ^a^	C_8_H_8_O_4_	167.0343	167.0343 (10.91), 152.0103 (100), 123.0154 (14.81), 95.0123 (9)	7.02	−3.903	1, 2
4	caffeic acid ^a^	C_9_H_8_O_4_	179.0337	179.0337 (17.89), 135.0435 (100)	4.79	−0.105	2, 3, 4
5	quinic acid	C_7_H_12_O_6_	191.0548	19.0548 (100), 173.0445 (1.81), 127.0384 (4.14), 85.0277 (22.37)	4.36	−6.811	1, 2, 3, 4
6	ferulic acid ^a^	C_10_H_10_O_4_	193.0501	193.0501 (19.29), 178.0263 (67.83), 149.0597 (20.28), 134.0360 (100)	5.77	−2.061	1, 2, 3, 4
7	isoferulic acid	C_10_H_10_O_4_	193.0493	193.0493 (100), 178.0260 (3.09), 161.0230 (17.21), 134.0360 (7.30)	11.50	0.055	1, 2, 3, 4
8	gentisic acid-O-hexoside	C_13_H_16_O_9_	315.0721	315.0721 (40.05), 153.0178 (74.49), 135.0072 (4.35), 109.0278 (100), 101.02281 (1.44), 65.0380 (7.62)	2.89	−0.144	1, 2, 3, 4
9	chlorogenic (5-caffeoylquinic) acid ^a^	C_16_H_18_O_9_	353.0887	353.0887 (2.42), 191.0553 (100), 85.0280 (7.41)	4.45	2.676	2, 3, 4
10	4-caffeoylquinic acid	C_16_H_18_O_9_	353.0874	353.0874 (30.06), 191.0558 (43.79), 179.0339 (69.67), 173.0445 (100), 135.0439 (58.77), 93.0331 (20.06)	4.79	−1.148	1, 2, 3, 4
11	ferulic acid 4-*O*-hexoside	C_16_H_20_O_9_	355.1039	193.0499 (100), 178.0264 (15.78), 149.0599 (9.40), 134.0361 (36.33)	5.74	1.280	1, 2, 3
12	syringic acid O-hexoside	C_15_H_20_O_10_	359.0974	359.0974 (6.71), 197.0449 (100), 182.0217 (19.01), 166.9986 (4.32), 153.0549 (15.22), 138.0309 (29.18), 123.0073 (25.01)	2.50	−2.673	2, 3, 4
13	3,4-dicaffeoylquinic acid ^a^	C_25_H_24_O_12_	515.1185	515.1185 (100), 353.0859 (7.33), 191.0554 (30.60), 179.0342 (66.02), 173.0450 (68.67), 161.0232 (8.19), 135.0437 (76.89), 93.0331 (10.24)	11.13	−1.882	1, 2, 3, 4
14	3,5-dicaffeoylquinic acid	C_25_H_24_O_12_	515.1179	515.1179 (10.48), 353.0880 (93.64), 191.0552 (100), 179.0340 (52.64), 135.0438 (56.36), 85.0282 (5.71)	11.40	−3.182	1, 2, 3, 4
15	4,5-dicaffeoylquinic acid	C_25_H_24_O_12_	515.1198	515.1198 (94.76), 353.0877 (51.76), 191.0554 (25.47), 179.0340 (58.14), 173.0445 (100), 161.0237 (4.88), 135.0438 (68.35), 93.0331 (21.78)	12.58	0.487	1, 2, 3, 4
**Rotenoids**							
16	boeravinone B	C_17_H_12_O_4_	311.0559	311.0559 (100), 283.0600 (6.92), 265.0504 (16.52), 237.0547 (4.88), 209.0599 (5.36), 147.0436 (4.22), 133.0278 (10.06), 109.0279 (3.63)	17.02	0.925	2
**Flavonoids**							
17	eupalitin	C_17_H_14_O_7_	329.0664	329.0664 (74.08), 314.0430 (100), 299.0194 (37.46), 271.0247 (49.23), 199.1331 (3.16), 171.0431 (0.82), 151.0024 (0.84), 112.9837 (3.08)	22.85	−0.717	1, 2, 3, 4
18	Isovitexin ^a^	C_21_H_20_O_10_	431.0984	431.0984 (100), 341.0669 (30.19), 311.0559 (70.53), 283.0606 (23.32), 269.0445 (2.52), 239.0713 (1.82), 183.5785 (1.14), 161.0237 (1.43), 117.0330 (8.93)	9.61	0.116	1, 2, 3, 4
19	kaempferol 3-*O*-glucoside ^a^	C_21_H_20_O_11_	447.0925	447.0925 (100), 285.0381 (18.27), 284.0320 (56.91), 255.0296 (35.53), 227.0334 (24.93), 177.3569 (4.09)	10.75	−1.755	2, 3
20	Isoquercitrin ^a^	C_21_H_20_O_12_	463.0873	463.0873 (100), 301.0346 (38.07), 300.0271 (81.06), 271.0245 (41.29), 255.0289 (14.51), 227.0339 (2.46), 151.0019 (7.54), 107.0116 (1.86)	9.51	−1.855	1, 2, 3, 4
21	Hyperoside ^a^	C_21_H_20_O_12_	463.0877	463.0877 (100), 301.0346 (42.87), 300.0271 (80.57), 271.0247 (39.22), 255.0289 (14.90), 227.0333 (1.55), 151.0018 (6.40), 107.0124 (1.25)	9.82	−1.121	1, 2, 3, 4
22	kaempferol-3-*O*-rutinoside ^a^	C_27_H_30_O_15_	593.1507	593.1507 (100), 284.0320 (65.84), 285.0381 (29.78), 255.0290 (34.66), 227.0344 (23.68), 117.0336 (3.60)	10.33	−0.832	2, 3, 4
23	Rutin ^a^	C_27_H_30_O_16_	609.1476	609.1476 (100), 301.0348 (40.88), 300.0273 (59.06), 271.0248 (34.49), 255.0296 (17.07), 151.0020 (6.31), 107.0120 (1.65)	9.55	−0.390	1, 2, 3
**Fatty Acids**							
24	azelaic acid	C_9_H_16_O_4_	187.0965	187.0965 (45.53), 141.8659 (1.28), 125.0958 (100), 123.0799 (3.96), 97.0643 (6.19)	11.66	−5.731	1, 2, 3, 4
25	dodecenedioic acid (traumatic acid)	C_12_H_20_O_4_	227.1286	227.1286 (8.77), 183.1382 (100), 165.1273 (16.47)	20.33	−1.375	2, 3, 4
26	13-hydroxy-9,11-octadecadienoic acid	C_18_H_32_O_3_	295.2279	295.2279 (100), 277.2174 (17.71), 195.1384 (18.61), 113.0958 (1.43)	28.67	0.142	1, 2, 3, 4
27	9-hydroxy-?-octadecenoic acid	C_18_H_34_O_3_	297.2435	297.2435 (100), 279.2329 (7.28), 155.1070 (12.75)	29.81	−0.162	2, 4
28	15-hydroxy-9-oxo-10,12,14-octadecatrienoic acid	C_18_H_26_O_4_	305.1761	305.1761 (93.79), 287.1661 (7.98), 249.1497 (68.78), 205.1595 (8.20), 185.1176 (2.64), 135.0803 (100), 125.0959 (22.92)	24.58	1.335	2, 3, 4
29	14-hydroxy-9-oxo-11,13,15-octadecatrienoic acid	C_18_H_28_O_4_	307.1918	307.1918 (100), 289.1797 (9.94), 197.1184 (14.02), 185.1176 (69.09), 109.0646 (5.04)	21.35	1.131	1, 2, 3, 4
30	14-hydroxy-9-oxo-11,13,15-octadecatrienoic acid	C_18_H_28_O_4_	307.1915	307.1915 (25.91), 289.1819 (17.24), 235.1335 (100), 211.1334 (32.15), 209.1172 (30.11), 185.1173 (69.58), 137.0952 (3.28), 121.0645 (81.36), 97.0644 (54.92)	23.61	0.057	1, 2, 3
31	9,10-dihydroxy-octadecanoic acid	C_18_H_36_O_4_	315.2542	315.2542 (100), 297.2441 (2.49), 245.1134 (1.23), 141.1274 (1.74)	27.55	0.498	2, 3, 4
32	9,10,13-trihydroxy-11,15-octadecadienoic acid	C_18_H_32_O_5_	327.2179	327.2179 (100), 291.1970 (4.22), 229.1444 (11.72), 211.1335 (11.95), 171.1019 (17.29), 137.0964 (1.66), 97.0644 (1.76), 85.0279 (5.99), 57.0331 (1.61)	19.76	0.711	1, 2, 3, 4
33	9,12,13-trihydroxy-10,15-octadecadienoic acid	C_18_H_32_O_5_	327.2179	327.2179 (100), 309.2075 (0.88), 291.1970 (3.35), 229.1442 (9.90), 171.1016 (19.90), 137.0960 (2.44), 97.0644 (2.13), 85.0280 (8.19)	20.41	0.497	1, 2, 3
34	9,10,15-trihydroxy-12,15-octadecadienoic acid	C_18_H_32_O_5_	327.2178	327.2178 (100), 291.1971 (4.03), 239.1646 (13.19), 211.1326 (3.22), 197.1174 (25.96)	22.05	1.628	2, 4
35	11,12,15-trihydroxy-9,12-octadecadienoic acid	C_18_H_32_O_5_	327.2177	327.2177 (100), 309.2074 (4.59), 197.1174 (32.96), 183.0113 (21.35), 111.0803 (9.25)	22.90	0.039	2, 4
**Others**							
36	heptanol pentosyl-hexoside	C_18_H_34_O_10_	409.2081	409.2081 (100), 277.1660 (30.60), 233.0650 (1.43), 161.0438 (9.50), 131.0335 (12.33), 113.0228 (11.04), 101.0228 (32.73), 89.0229 (15.08), 71.0122 (39.57)	12.82	0.512	2
37	ursolic acid hexuronyl-hexoside	C_42_H_66_O_14_	793.4395	793.4395 (100), 631.3859 (10.19), 569.3862 (7.64), 455.3518 (2.06), 316.2431 (0.52), 175.0233 (0.71), 157.0130 (0.74), 113.0231 (8.27), 85.0280 (17.25)	2323	1.929	1, 2, 3, 4

^a^ compared to reference standard; 1-Ethyl acetate extract; 2-MeOH extract; 3-Dichloromethane extract; 4-Infusion with water.

## Data Availability

Data is contained within the article and Supplementary Materials.

## Supplementary Materials

The following are available online at https://www.mdpi.com/article/10.3390/antiox10122003/s1, supplementary material and methods (details of chromatographic analysis) are available online.
