# Supplementary material for: LC-MS/HRMS Analysis, Anti-Cancer, Anti-Enzymatic and Anti-Oxidant Effects of Boerhavia diffusa Extracts: A Potential Raw Material for Functional Applications
Source: Antioxidants (Basel). 2021 Dec 16;10(12):2003. doi: 10.3390/antiox10122003 (PMC8698501; doi:10.3390/antiox10122003)
Supplement: Supplementary file 1 [file antioxidants-10-02003-s001.zip › antioxidants-1486217-supplementary.pdf]

Supplementary Materials

Chemicals

Acetonitrile and formic acid for LC-MS, and methanol of analytical grade were purchased from Merck (Merck, Bulgaria). The reference standards used for the compounds identification were obtained as follows: protocatechuic acid, gentisic acid, vanillic acid, ferulic acid, kaempferol 3-*O*-rutinoside, isovitexin, isoquercitrin, hyperoside, and rutin, from Extrasynthese (Genay, France); caffeic acid and 3,4-dicaffeoylquinic acid were supplied from Phytolab (Germany); chlorogenic (5-caffeoylquinic) acid was purchased from Sigma-Aldrich (St. Louis, MO, USA).

Chromatographic separation

Separation was achieved on an ultra-high-performance liquid chromatography (UHPLC) system Dionex Ultimate 3000RSLC (ThermoFisher Scientific, Inc.) with reversed phase column Kromasil EternityXT C18 (1.8 µm, 2.1 × 100 mm) column maintained at 40°C. The binary mobile phase consisted of A: 0.1% formic acid in water and B: 0.1% formic acid in acetonitrile. The run time was 33 min. The following gradient was utilized: the mobile phase was held at 5% B for 1 min, gradually turned to 30% B over 19 min, increased gradually to 50% B over 5min, increased gradually to 70% B over 5 min, and finally increased gradually to 95% over 3 min. The system was then turned to the initial condition of 5% B and equilibrated over 4 min. The flow rate and the injection volume were set to 300 µL/min and 1 µL, respectively. The effluents were connected on-line with a Q Exactive Plus Orbitrap mass spectrometer where the compounds were detected (Zengin et al., 2019a).

High resolution mass spectrometry (HRMS)

Mass analyses were carried out on a Q Exactive Plus mass spectrometer (ThermoFisher Scientific, Inc.) equipped with a heated electrospray ionization (HESI-II) probe (ThermoScientific). The tune parameters were as follows: spray voltage 3.5 kV; sheath gas flow rate 38; auxiliary gas flow rate 12; spare gas flow rate 0; capillary temperature 320 °C; probe heater temperature 320 °C and S-lens RF level 50. Acquisition was acquired at Full-scan MS and Data Dependent-MS^2 modes. Full-scan spectra over the m/z range 100 to 1500 were acquired in negative ionization mode at a resolution of 70,000. Other instrument parameters for Full MS mode were set as follows: AGC target 3e6, maximum ion time 100ms, number of scan ranges 1. For DD-MS^2 mode, instrument parameters were as follows: microscans 1, resolution 17,500, AGC target 1e5, maximum ion time 50ms, MSX count 1, isolation window 2.0 m/z, stepped collosion energy (NCE) 20, 40, 70 eV. Data acquisition and processing were carried out with Xcalibur 4.2 software (ThermoScientific) (Zengin et al., 2019b)
